# Social determinants of depression and suicidal behaviour in the Caribbean: a systematic review

**DOI:** 10.1186/s12889-017-4371-z

**Published:** 2017-06-15

**Authors:** Catherine R Brown, Ian R Hambleton, Natasha Sobers-Grannum, Shawn M Hercules, Nigel Unwin, E Nigel Harris, Rainford Wilks, Marlene MacLeish, Louis Sullivan, Madhuvanti M Murphy, The U.S. Caribbean Alliance for Health Disparities Research Group (USCAHDR), M Alvarado, N Bennett, A Bidulescu, CR Brown, T Ferguson, D Francis, IR Hambleton, EN Harris, C Hassell, AJM Hennis, SM Hercules, C Howitt, M Mac Leish, MM Murphy, TA Samuels, N Sobers-Grannum, L Sullivan, N Unwin, R Wilks, L Williams, N Younger-Coleman

**Affiliations:** 1 George Alleyne Chronic Disease Research Centre, Bridgetown, Barbados; 2 The University of the West Indies, Cave Hill, Barbados; 3 The University of the West Indies, Kingston, Jamaica; 4 Sullivan Alliance, Alexandria, VA USA; 5 McMaster University, Hamilton, Canada

## Abstract

**Background:**

Depressive disorder is the largest contributor to years lived with disability in the Caribbean, adding 948 per 100,000 in 2013. Depression is also a major risk factor for suicidal behaviour. Social inequalities influence the occurrence of depression, yet little is known about the social inequalities of this condition in the Caribbean. In support of the 2011 Rio Political Declaration on addressing health inequities, this article presents a systematic review of the role of social determinants on depression and its suicidal behaviours in the Caribbean.

**Methods:**

Eight databases were searched for observational studies reporting associations between social determinants and depression frequency, severity, or outcomes. Based on the PROGRESS-plus checklist, we considered 9 social determinant groups (of 15 endpoints) for 6 depression endpoints, totalling 90 possible ways (‘relationship groups’) to explore the role of social determinants on depression. Studies with ≥50 participants conducted in Caribbean territories between 2004 and 2014 were eligible. The review was conducted according to STROBE and PRISMA guidelines. Results were planned as a narrative synthesis, with meta-analysis if possible.

**Results:**

From 3951 citations, 55 articles from 45 studies were included. Most were classified as serious risk of bias. Fifty-seven relationship groups were reported by the 55 included articles, leaving 33 relationship groups (37%) without an evidence base. Most associations were reported for gender, age, residence, marital status, and education. Depression, its severity, and its outcomes were more common among females (except suicide which was more common among males), early and middle adolescents (among youth), and those with lower levels of education. Marriage emerged as both a risk and protective factor for depression score and prevalence, while several inequality relationships in Haiti were in contrast to typical trends.

**Conclusion:**

The risk of bias and few numbers of studies within relationship groups restricted the synthesis of Caribbean evidence on social inequalities of depression. Along with more research focusing on regional social inequalities, attempts at standardizing reporting guidelines for observational studies of inequality and studies examining depression is necessitated. This review offers as a benchmark to prioritize future research into the social determinants of depression frequency and outcomes in the Caribbean.

**Electronic supplementary material:**

The online version of this article (doi:10.1186/s12889-017-4371-z) contains supplementary material, which is available to authorized users.

## Background

The Global Burden of Disease study has ranked depressive disorders as the largest contributor to years lived with disability (YLD) in the Caribbean since 1990, with these conditions adding 948 YLD per 100,000 in 2013 [1, 2]. Among 15–49 year olds, this represents 10% of all YLD [1, 2]. Suicide, a last-resort outcome of depression, occurs at a global rate of 11.4 per 100,000, though stigma-associated underreporting underestimates the true value [3]. While suicide represents the second leading cause of death among 15–29 year olds globally, an important risk factor such as depression often goes undiagnosed or untreated [3]. Even more, undiagnosed or untreated depression is a risk factor for increased adverse outcomes of many chronic and acute illnesses [4–8].

Social experiences throughout the life course influence the occurrence of depressive disorders and subsequent adverse outcomes [9]. For instance, income inequality, particularly in wealthy countries, is associated with a higher prevalence of mental disorders, and the degree of socioeconomic disadvantage is proportionate to the risk of developing such a disorder [10]. Moreover, when socioeconomic inequalities are perpetuated through generations, inequalities are further entrenched in depressive disorders over time [10]. Examining whether there are differences among particular groups, and determining their basis, can guide policy towards improving outcomes. The World Health Organization (WHO) Commission on the Social Determinants of Health (CSDH) has highlighted the role of health research in understanding health inequalities and inequities, and through the 2011 Rio Political Declaration, countries have committed to monitoring, understanding and addressing health inequities [11, 12].

Globally, research on social determinants of depression and its outcomes is limited mostly to primary studies. Observational studies from India, USA, UK, and Europe report that older age, female gender, lower education, and poor economic status are associated with depression [13–17]. However, systematic reviews are limited in scope as they tend to focus narrowly on economic disadvantage, showing that the poor and disadvantaged suffer disproportionately from common mental disorders and their adverse consequences [9, 18–20]. Other social determinants as well as other regions warrant exploration to illustrate whether regional or country-level contexts have a role to play [9]. To date, there has been no published systematic assessment of evidence on the social determinants of depression among Caribbean populations. This systematic review is guided by the analytical framework used to examine the social determinants of specific conditions by the WHO CSDH [21]. This review uses a simplified version of the framework to answer the primary research question: what is the distribution, by known social determinants of health, of the frequency, severity, and adverse outcomes of depression among populations living in the Caribbean?

## Methods

Full details of the review methodology are available in the study protocol (see Additional file 1). The protocol was guided by an initial scoping review of depression, a previous systematic review of social determinants of diabetes [22], and concurrent systematic reviews of breast and prostate cancer.

### Eligibility criteria

Observational studies of any design were sought that reported relationships between a social determinant and depression frequency (incidence, prevalence), depression severity (score on any depression scale) or depression outcomes (suicide ideation, parasuicide, suicide). These particular outcomes were selected based on an initial scoping review of depression which showed a majority of research to examine these variables. Articles published between January 2004 and December 2014 in the dominant Caribbean languages (English, Spanish, French, and Dutch) were sought from 32 Caribbean territories. This 10-year period was selected as the study is taking place within the context of a major review of regional and national policy responses in the Caribbean to chronic non-communicable diseases [23]; therefore the findings have relevance to the current situation and could inform policy response.

Included studies drew upon samples from either the general population or healthcare facility catchments. Age restrictions were not used, but sample sizes <50 were excluded as unlikely to be fully representative of underlying populations. The selection of social determinants was guided by the extension of the PRISMA statement for the transparent reporting of systematic reviews and meta-analyses with a focus on health equity, which recommends the “PROGRESS-Plus” checklist [24]. This acronym checklist refers to a core list of social determinants, namely: place of residence, race or ethnicity (alternatively culture or language), occupation, gender, religion, education, socio-economic position (SEP), social capital, plus other social determinants that might be of interest [24]. For this study, ‘age’ was included a ‘Plus’ to this listing.

### Search strategy, study selection, data extraction

The databases searched were: MEDLINE (via Pubmed); EMBASE (via Ovid); SciELO; PsycInfo (via EBSCO); CINAHL (via EBSCO); CUMED, LILACS, and IBECS (via WHO Virtual Health Library) [25–30]. The final search was conducted in February 2015. The search strategies are detailed in a supplementary file (see Additional file 2). Search results were maintained in Endnote reference manager software [31].

Study selection and data abstraction were undertaken in duplicate by two independent reviewers (CRB, SMH); any inconsistencies were resolved by a third reviewer (MMM). Study selection was conducted in two stages. First, titles and abstracts were screened to identify potentially relevant articles; second, full-text screening of potentially relevant articles was conducted to identify articles for inclusion in the review. If inadequate information was available for decision-making in the first stage, the article automatically progressed to full-text review. In addition to those excluded, 8 articles were either inaccessible or awaiting publication [32–39]. With guidance by the STROBE statement on strengthening the reporting of observational studies in epidemiology and the PRISMA-Equity statement [40, 41], an electronic data abstraction form was created in REDCap database [42] (see Additional file 1).

### Risk of bias assessment

Risk of bias was assessed according a tool adapted from STROBE and Cochrane ACROBAT-NRSi guidelines (see Additional file 1) [40, 43]. Bias was assessed in 5 domains at the relationship level: confounding (was control for known and potential confounders adequate?), participant selection (is the sample representative of the target population?), missing data (is the data reasonably complete?), outcome measurement (is a social determinant or disease endpoint appropriately measured?), selective reporting (is a relationship selectively reported?). Studies were classified as having serious, moderate, low, or unclear risk of bias. To accommodate the different tools and methods used to identify depressive disorders, the use of a validated tool and the involvement of clinical expertise were considered to be necessary features of the screening process. If a measurement tool was not validated or if a clinician was not involved in the screening, the relationship was classified as “high-risk” under the outcome measurement domain. Two reviewers (CRB, MMM) made an independent judgement on the overall risk of bias of each included article, considering equal importance of each domain and the likely direction and magnitude of the bias from each domain. Discrepancies were discussed by the two reviewers to achieve consensus.

### Synthesis of results

The review was planned as a narrative synthesis with supplementary meta-analysis if possible. Key study details are presented, followed by a description of each association between a social determinant and either a measure of depression frequency, severity, or outcome. The number and type of inequality relationships were summarized in an ‘evidence gap map’ – a visual tool to highlight the current evidence on the social determinants of depressive disorders in the Caribbean and as a guide for focusing future research [44] Given the methodological heterogeneity of the study settings and their measurement tools, meta-analysis was not conducted.

## Results

### Summary of included studies

**Fig. 1:**
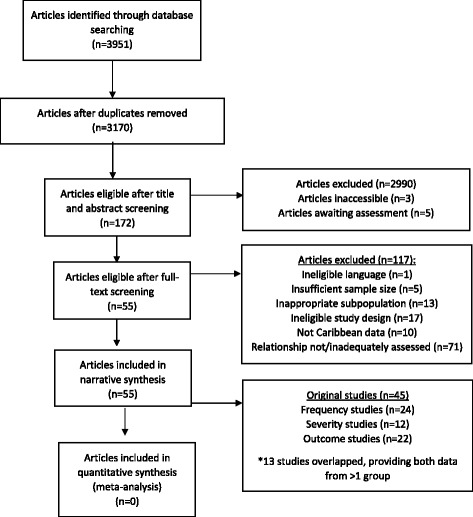
Flowchart of search strategy and article selection

Figure 1 presents a flowchart of articles identified, excluded, and included. Additional file 3: Table S1 describes characteristics of the 55 included articles, from 45 unique studies which examined one or more inequality relationships. Of these 55 articles, 29 reported on depression frequency, 15 reported on depression severity, and 18 reported on depression outcomes (12 articles overlapped examining measures from two endpoint groups). A total of 15 social determinants were examined. Depression frequency was reported as a prevalence in all articles. Studies were conducted in English-speaking (Bahamas, Barbados, Grenada, Guyana, Jamaica, St. Kitts & Nevis, St. Lucia, St. Vincent, Trinidad and Tobago); French-speaking (Haiti, Martinique); Spanish-speaking (Cuba, Dominican Republic, Puerto Rico); and Dutch-speaking (Suriname) Caribbean territories. Majority of articles originated from Cuba (*n* = 15) and Jamaica (*n* = 15).

**Fig. 2:**
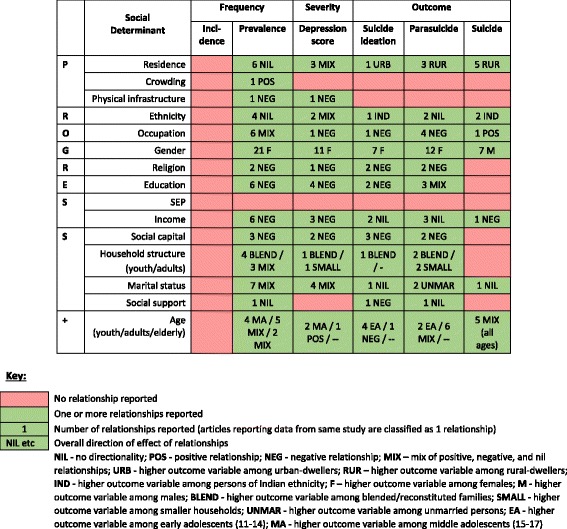
Summary of 214 unique inequality relationships among 55 included articles [45–86, 99, 100–111]

Figure 2 illustrates the distribution of inequality relationships among the included articles. Across the nine categories of social determinants, there were a total of 15 social determinants and 6 review endpoints, totalling 90 inequality relationship groups that could have been reported. Fifty-seven (63%) of these relationship groups were reported by the 55 included articles, leaving 33 relationship groups (37%) without an evidence base. There were 222 inequality relationships reported: 86 on depression frequency, 15 on depression severity, and 121 on depression outcomes. When articles reporting data from same study were considered and removed, the number of inequality relationships fell to 214: 82 reporting depression frequency, 13 reporting depression severity, and 119 reporting depression outcomes. While most relationship groups were explored, the quantity of inequality relationships *within* each group was limited.

### Risk of bias of included studies

**Fig. 3:**
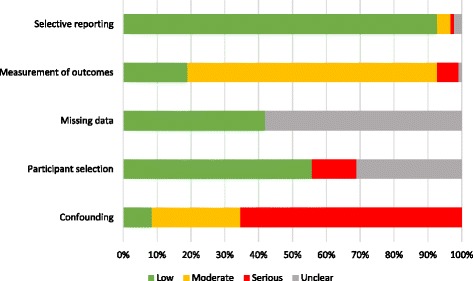
The proportion of risk of bias classifications of the 222 relationships among each of the risk of bias domains

A summary of the overall risk of bias classification assigned to each of the 55 articles is presented in Additional file 4: Table S2. Classifications specific to each article in each of the five domains are described in an extended table, Additional file 5: Table S3. Of the 55 articles, 11 were classified as moderate-risk, 31 were classified as serious-risk, 8 were classified as unclear-risk, 3 were classified as serious/moderate-risk, and 2 were classified as serious/unclear-risk. Figure 3 details the proportion of relationship classifications within each of the risk of bias domains. Overall, lack of adjustment for potential confounding was the main contributor to an increased risk of bias, followed by non-disclosure or inadequate handling of missing data. This collective high risk of bias of the included studies must be taken into consideration when interpreting results.

### Results of inequality relationships

The amount of inequality relationships, stratified by social determinant, varied greatly – from 61 examining gender, to 1 examining crowding. The results of the social determinants which contributed the most relationships – gender, age, residence, marital status, and education - are detailed below. Descriptions for the remaining social determinants are located in a supplementary file, Additional file 6.

### Gender

Gender was examined in 61 inequality relationships (58 unique) across 47 articles: depression prevalence (*n* = 23), depression score (*n* = 11), suicidal ideation (*n* = 7), parasuicide (*n* = 12), suicide (*n* = 8). Among these, 12 were classified as having moderate risk of bias, 38 as having serious risk of bias, and 11 as having unclear risk of bias. Across depression prevalence, depression score, suicidal ideation and parasuicide, females outnumbered males with only minor exceptions: one study from Haiti found a slightly higher prevalence (92% vs 86.5%) and depression scores (x = 23.4 vs x = 21.1) among males [45]; and three studies showed slightly more parasuicide in males in Martinique and Puerto Rico [46, 47, 48]. Suicide, however, unanimously occurred more frequently by males across several countries [49–56].

### Age

Age was examined in 32 inequality relationships across 26 articles: depression prevalence (*n* = 11), depression score (*n* = 3), suicidal ideation (*n* = 5), parasuicide (*n* = 8), suicide (*n* = 5). Among these, 10 were classified as having moderate risk of bias, 17 as having serious risk of bias, and 5 as having unclear risk of bias. Of those studies examining depression in adolescents specifically, most found the highest prevalence and depression scores among 16 and 17 year olds [57–60]. However, suicidal ideation and parasuicide were more prevalent among younger adolescents aged <16 years than those older [46, 48, 61, 62]. Adult studies examining depression prevalence reported varied results, but the single adult study examining depression score found older age to be associated with increased scores for both genders in Haiti [63]. The sole adult study examining suicidal ideation in adults found Puerto Ricans aged ≤50 years to be more likely to perform this than those aged >64 years (OR 1.71, 95%CI 1.39–2.65) [48]. Lower rates of parasuicide and suicide were also reported among elderly, but without a definitive peak among the younger age groups [47–51, 53, 54, 64, 65]. An exception is one registry-based study which reported a peak suicide rate of 28.7 per 100,000 in Cubans aged >74 years, with rates decreasing with age [50].

### Residence

Residence was examined in 22 inequality relationships (18 unique) across 20 articles: depression prevalence (*n* = 7), depression score (*n* = 5), suicidal ideation (*n* = 1), parasuicide (*n* = 3), suicide (*n* = 6). Among these, 6 were classified as having moderate risk of bias, 14 as having serious risk of bias, and 2 as having unclear risk of bias. No associations were found between residence and depression prevalence [66–72]. Depression score was examined on a country-level. Two studies examining elderly in three countries found depression scores to be higher in the Dominican Republic, followed by Cuba, then Barbados [73, 74]. One study reported higher depression scores among Jamaican adolescents than adolescents in St. Vincent, St. Kitts and The Bahamas [75–77]. Jamaican adolescents living in urban areas reported a higher prevalence of suicidal ideation among than those living in rural areas [78]. This is to contrast what is seen for parasuicide and suicide, which occurred more often in rural areas [46–53]. One study examined suicide in a country-level; highest rates were found in in Guyana (22.4 per 100,000) and Suriname (15.3), followed by Trinidad (7.0), Cuba (4.0) and Puerto Rico (3.6) [54].

### Marital status

Marital status was examined in 16 inequality relationships (15 unique) across 12 articles: depression prevalence (*n* = 8), depression score (*n* = 4), suicidal ideation (*n* = 1), parasuicide (*n* = 2), suicide (*n* = 1). Among these, 3 were classified as having moderate risk of bias, 12 as having serious risk of bias, and 1 as having unclear risk of bias. Overall findings across these variables were inconclusive. While a higher depression prevalence was found among persons not in a relationship in Cuba, Barbados, and Trinidad [73, 79, 80], studies from Jamaica found higher prevalence among persons who are married versus unmarried [81, 82]. The same applies for depression score: higher scores were reported among unmarried persons in Haiti and Jamaica [63, 81], but also among married persons in another two studies from Jamaica and Puerto Rico [82, 83]. Two studies examining parasuicide reported married persons to be less likely to report parasuicide [47, 48].

### Education

Education was examined in 15 inequality relationships across 13 articles: depression prevalence (*n* = 6), depression score (*n* = 4), suicidal ideation (*n* = 2), parasuicide (*n* = 3). Among these, 1 was classified as having moderate risk of bias, 13 as having serious risk of bias, and 1 as having unclear risk of bias. Most studies examining the frequency and score of depression and suicide ideation demonstrated a higher prevalence/score among persons with less education or maternal education [48, 58, 70, 79, 81, 82, 84–86]. In Trinidad, persons with primary education only are nearly three times as likely to have depression than those with secondary or higher education (OR 2.7, 95%CI 1.4–5.1) [84]. Suicidal ideation was twice as common among Puerto Ricans with <12 years of overall education when compared to those with >3 years of college education (OR 2.21, 95%CI 1.31–3.74) [48]. The single Haitian study examining education contrasted these trends, which found higher depression scores among more educated females [63].

## Discussion

### Summary of evidence

This systematic review has examined the extent of evidence on the influence of social determinants of health on depression frequency, severity, and adverse outcomes in the Caribbean. Fifty-five articles from 45 separate studies were included. With 90 possible ways (relationship groups) of exploring the role of social determinants on depression, 222 relationships were reported looking at 57 distinct relationship groups, leaving 33 relationship groups (37%) without an evidence base.

Overall, most of our findings mirror global trends [87]. Depression frequency, depression severity, and suicidal behaviour were higher among females (with the exception of suicide being more common in males); persons with lower education, income, and occupation levels; those participating in less religious activity; and those with less social capital and support. The connection between depression and social inequity is not a new phenomenon as disadvantaged groups have been shown to place individuals at a higher risk of developing and dying from this condition [9, 18, 87]. For instance, the occurrence of common mental disorders is shown to be associated with low educational attainment, material disadvantage, unemployment, and social isolation [19, 87]. This social class gradient is more marked among females than males [9], perhaps partly explaining the heavy female burden of mental disorder.

Important to note, however, are the geographic and cultural idiosyncrasies that can affect mental health trends, making the examination of depression highly context-specific [9, 87, 88]. For instance, evidence from Haiti contrasted typical global trends. The two Haitian studies in this review found a higher depression prevalence and scores in males than females, higher depression scores among those with more education than less, and higher depression scores with increasing age than decreasing age [45, 63]. Haiti’s poor economic situation, exacerbated by recent natural disasters, could be an explanatory factor for increases in scores in these groups as violence and childhood neglect, both associated with current and later-life depression, increase with decreasing economies [45, 88]. Specifically, increased depression scores in more highly educated Haitian women could result from a cognitive dissonance between an optimistic aspiration of professional employment and the stark reality of the country’s limited employment opportunities [63, 89].

Interestingly, there is a paucity of research (*n* = 3) from Suriname and Guyana, two countries which not only have predominant portions of East Indians making up their ethnography, but also some of the highest rates of suicide worldwide [90–92]. Suicide has permeated the East Indian culture, often glorified as courageous and a means to avoid shame and disgrace [93]. Whether the impact of ethnicity on depression/suicidal behaviour is grounded in deep cultural customs or perhaps social disparities woven into ethnic status of these countries is an area which needs further investigation.

Social factors act as buffers throughout stressful circumstances (such as living in a low SEP) by offering emotional, informational, or instrumental resources [9, 94] This is evidenced in the negative associations found with social capital, social support and social household structure. For this reason, our overall inconclusive finding for marital status is unexpected as marriage is generally thought to offer improved social capital and support [87, 95, 96]. Furthermore, marital status in Jamaica was a risk factor for depression prevalence and severity, while studies in other Caribbean territories found it to be protective or have no association. This begs to consider the quality and context of marriages in Caribbean countries, as particular factors such as relationship quality, extended family support, and ability to cope with marital stress and child rearing likely confound relationships between marital status and depression occurence [88, 95]. The interaction between social determinants themselves is an important consideration. As in this example, the relationship between marital status and depression may be moderated by the level of social support outside of the marriage. More specifically, the inverse relationship between education and depression is certainly moderated by the setting, as can be seen in evidence in Haiti versus other Caribbean countries.

Contradictory reports might also be due to differing methodology. Assessing risk of bias of depression studies was challenging due to lack of detail and explicitness of the measurement of depression and suicidal behaviour. For example, some studies failed to state the depression scale used (was it evidence-based?) or who delivered the scale (was this individual trained?). In attempts at accommodating these elements, risk of bias methodology considered ‘validation of measurement tools’ and ‘clinician involvement’ in the measurements of variables. These added caveats emphasize the significance of and need for a standardised tool for assessing risk of bias of subjective measures such as depression, as a gold standard does not currently exist. It is recommended that researchers in this field give sufficient detail on the methods of assessment to allow for more objectivity in reporting.

The relationship between depression and social factors can be bidirectional; while depression perpetuates reduced education, employment and income by interfering with ones capacity to function in productive roles, this social decline can itself increase the development of depression and exacerbate  its outcomes [9]. Regardless, the median rate for treated depression is only 50%, of which only a small proportion of this treatment is considered adequate [87]. Treatment deficiencies could be improved more efficiently by considering the social inequities that put certain groups at higher risk.

How best to fill this evident research gap is an important consideration [97]. While improving the quality of studies is a recommendation across the board, it is less obvious whether research should be prioritized to focus on areas with no research (Fig. 2, red boxes) or to work towards improving the existing low-level evidence base (Fig. 2, green boxes). Currently, regional focus on mental health is weak, and many mental health systems are behind in their efforts at decentralization and prevention services [98]. Examining the social inequalities help, at the very least, to justify prioritization of addressing mental deprivation and inequality in the Caribbean.

### Limitations

There is an unavoidable limitation in social determinant studies: interrelationships among the social determinants themselves which act as confounders. Caribbean evidence is limited in its quality and distribution across social determinants. The majority of articles were classified as having high risk of bias, mostly because of failure to adjust for important potential confounders, but also due to the variation in sampling and screening instruments. Additionally, inconclusive findings within many relationship groups could at least be partially due to a small number of studies available within each group. There is also the potential for missing data from individual studies, possibly due to the sensitivity of the disease and its outcomes and associated non-reporting. The Caribbean has been considered as one region in this review, masking what is likely to be important country-level variation in the relative importance of social determinants. Country-level information on depression screening and access to treatment are important potential confounders that were not assessed. No explicit searching was conducted for grey literature due to limited resources.

## Conclusions

Of 15 social determinants examined, gender, age, residence, marital status, and education contributed the most inequality relationships, with gender accounting for 27% of all relationships. The WHO CSDH has emphasised the importance in understanding health inequalities, and the Caribbean has pledged to address these [11, 12]. Along with more research focusing on regional social disparities in the Caribbean, attempts at standardizing observational reporting guidelines for observational studies of inequality is necessitated. This review offers as a benchmark to prioritize future research into the social determinants of depression frequency, severity, and outcomes in the Caribbean.

## Additional files

Additional file 1: Study Protocol, which details the study protocol for the systematic review. (PDF 2252 kb)

Additional file 2: Search Strategy, which details the search strategies of the database. (PDF 472 kb)

Additional file 3: Table S1. Characteristics of 55 articles from the Caribbean region describing the social distribution of depression [45–86, 99, 100–120]. (XLSX 41 kb)

Additional file 4: Table S2. Risk of bias among 222 inequality relationships from 55 included articles [45–86, 99, 100–111]. (XLSX 14 kb)

Additional file 5: Table S3. Extended risk of bias classification table of 55 included articles, to supplement Table S2, which depicts the risk of bias classification of each of the five domains for individual relationships of each article. (XLSX 21 kb)

Additional file 6: Supplementary Narrative of Results of Inequality Relationships, which describes additional results for remaining social determinants not discussed narratively in the main paper. (DOCX 54 kb)

## Abbreviations

CINAHL: Cumulative Index of Nursing and Allied Health Literature
CSDH: Commission on the Social Determinants of Health
CUMED: Cuba Medicina
EMBASE: Excerpta Medica Database
IBECS: Índice Bibliográfico Español en Ciencias de la Salud
LILACS: Latin American and Caribbean Health Sciences
MEDLINE: Medical Literature Analysis and Retrieval System Online, or MEDLARS Online
NCD: Non-communicable disease
SciELO: Scientific Electronic Library Online
STROBE: Strengthening the Reporting of Observational studies in Epidemiology SEP Socioeconomic Position
USCAHDR: United States Caribbean Alliance for Health Disparities Research Group
WHO: World Health Organization
