# Supplementary material for: Social determinants of depression and suicidal behaviour in the Caribbean: a systematic review
Source: BMC Public Health. 2017 Jun 15;17:577. doi: 10.1186/s12889-017-4371-z (PMC5472962; doi:10.1186/s12889-017-4371-z)
Supplement: Supplementary file 1 — Study Protocol, which details the study protocol for the systematic review. (PDF 2252 kb) [file 12889_2017_4371_MOESM1_ESM.pdf]

Study Protocol

Title

Health inequities of the distribution and adverse outcomes of depression in populations living in the Caribbean: a systematic review.

Background

The World Health Organization estimates that 350 million people worldwide suffer from depression, ranking it as the leading cause of disability. (1) Depression represents a serious medical condition, with suicide being its worst outcome. (1) And although effective treatments are known, less than 50% of sufferers receive this assistance, owing largely to a lack of resources, lack of trained health care providers, misdiagnosis, and social stigma.(1) The *2011 World Conference on Social Determinants of Health* and its subsequent *Rio Political Declaration on Social Determinants of Health* rightfully recognize the critical role that social determinants play in the distribution of noncommunicable diseases such as depression, as well as government commitment to improving sustainable development and health equity using the social determinants approach. (2) This calls for the ethical and public health challenge of identifying and addressing health inequities between populations and groups, such that the World Health Organization's *Global Action Plan on Prevention and Control of Noncommunicable Diseases 2013-2020* can be utilized. (3,4) Depression is the largest contribution to years lived with disability and is a significant contribution to disability-adjusted life years within the Caribbean. (5,6) Despite its heavy burden on regional morbidity rates, there exists no published systematic review of research conducted in the Caribbean that examines the social determinants of depression. Such a review is necessary as it would not only inform regional preventive strategies for depression and its complications, but also identify areas for further research.

Systematic Review Framework

The planning of this systematic review was guided by the analytical framework used to examine the social determinants of specific conditions by the WHO Commission on the Social Determinants of Health. (3) The framework has five levels and three dimensions, as shown in Figure 1 below. The

Commission's starting point to using this framework was to examine differential health outcomes by markers of social and economic status (such as gender, ethnicity, education, and occupation), and then to look upstream to investigate where these differences originate. After analyzing the determinants in this way, contributors to the WHO Commission then examined potential interventions to address the determinants, and suggested indicators to be measured in order to assess the success of those interventions (the 'intervene' and 'measure' dimensions in Figure 1).

Figure 1: Analytical Framework for Priority Public Health Conditions used by the WHO Commission on Social Determinants. Taken from: Blas E, Kurup AS, editors. Equity, social determinants, and public health programmes [Internet]. World Health Organization: World Press; 2010. (7)

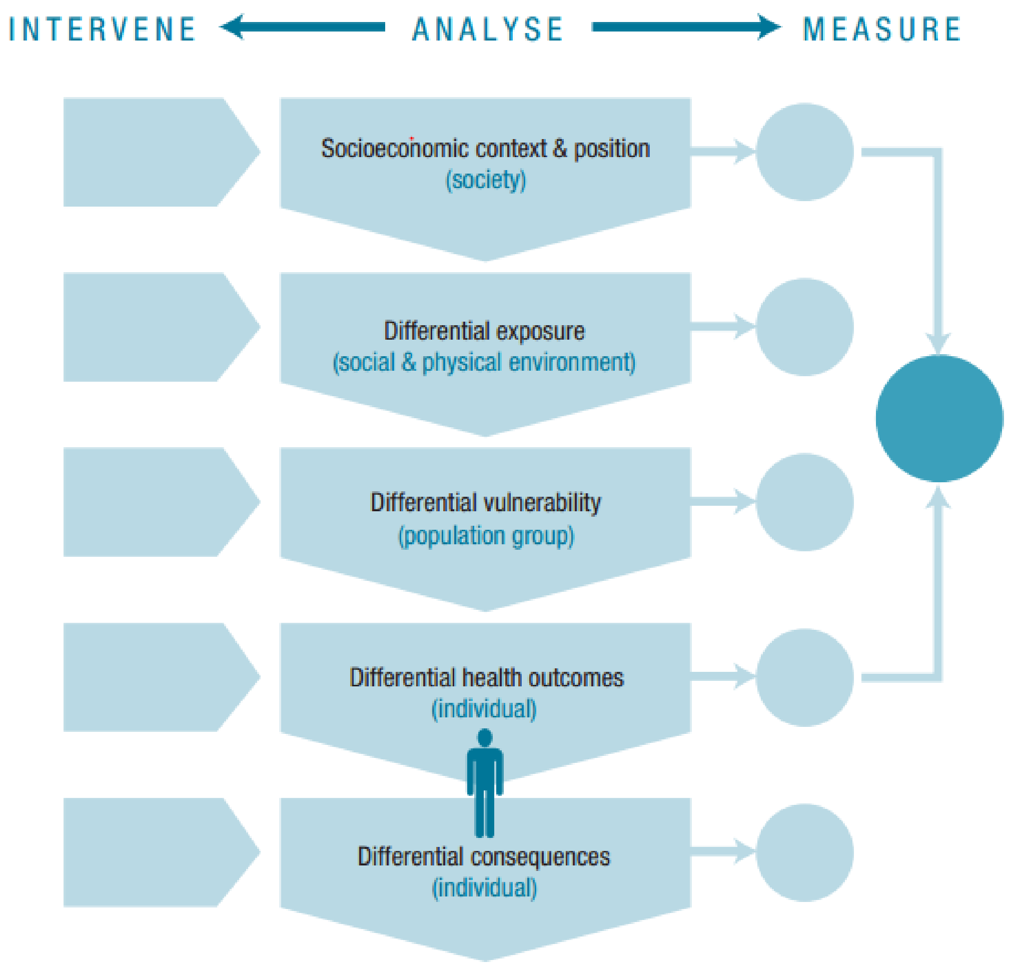

Reviewing the literature across the five levels and three dimensions is beyond the scope of a single review. Thus, our aim of this review is to provide a solid foundation for further work on health inequities of depression in the Caribbean by reviewing the social distribution of the prevalence and incidence, and major outcomes ('consequences') of depression. Distribution of risk factors ('vulnerabilities') for

depression is not measured in this review as there lacks clarity on their certainty due to the complexity of the disease, to which the World Health Organization aims to develop in the future. (8)

There is a clear rationale underpinning the chosen inclusion criteria for this review. All ages are selected for the population to keep the review as broad as possible. A sample size limit of $\geq$50 participants or respondents is used as it is expected that studies with a small sample size will be less likely than larger studies to be representative of the population. The study types included were all observational and the review is aimed at assessing the distribution and adverse outcomes of depression. Interventions are included as well, but not to assess intervention success, but rather to allow their baseline data to inform in a cross-sectional manner. The outcomes and risk factors to be assessed (see Table 1) were selected specifically to ensure that the items were broadly scoped to capture as many studies as possible. The social determinants selected for the inclusion criteria (see Table 1) were guided by the extension of the PRISMA statement for reporting systematic reviews with a focus on health equity. (9)

Review Question

Primary Question: What is the distribution, by known social determinants of health, of the incidence, prevalence, and adverse outcomes of depression in populations living in the Caribbean?

Secondary Question: What are the implications of this distribution for reducing and preventing further health inequities within the Caribbean region?

Methods

Inclusion & Exclusion Criteria

Inclusion Criteria:

- Participants/respondents resident in the Caribbean region, inclusive of the following countries: Anguilla, Antigua and Barbuda, Aruba/Bonaire/Curacao, The Bahamas, Barbados, St. Bart's, Belize, Cayman Islands, Cuba, Dominica, Dominican Republic, St. Eustatius, French Guiana, Grenada, Guadeloupe, Guyana, Haiti, Jamaica, St. Kitts and Nevis, St. Lucia, St. Martin, St. Maarten, Martinique, Montserrat, Puerto Rico, St. Vincent and The Grenadines, Saba, Suriname, Trinidad & Tobago, Turks & Caicos, and the Virgin Islands (US and British)
- Published observational studies.

- Studies which define depression according to the DSM-5 criteria for "Depressive Disorders" or through any depression screening tool
- Sample size $\geq$50
- Age of study participants: all ages
- Studies describing the distribution of $\geq$1 factors in rows (A) or (B) in the Table 1 below by $\geq$1 social factors in row (C).

Table 1: Key variables to be abstracted and collected.

Group	Factor Categories	Factors Being Examined
**A**	Disease measurements	incidence, prevalence
**B**	Outcomes	depression score, suicide ideation, suicide, para-suicide, all-cause mortality
**C**	Social distribution	age, race/ethnicity, gender, language, education, occupation, income/wealth, culture, religion, social capital, social support, residence, infrastructure, healthcare systems

Exclusion Criteria:

- Intervention studies, narrative review papers, commentaries, case series, qualitative studies and single case reports.
  - Note that while case series and qualitative papers are excluded for purposes of data extraction, information from these types of studies will be used to inform the discussion.
- Unpublished observational studies.
- Studies in which describe the relationships of interest within sub-populations that are not representative of the general population (eg: patients in renal failure).
- Literature on Caribbean diaspora (as opposed to populations living within the Caribbean).
- Literature that is written in any other language than English, Dutch, Spanish and French.

- Non-human studies.
- Sample size <50.

Search Strategy

The search strategies for breast cancer and prostate cancer according to the specifications of the Pubmed search engine are detailed in Appendix A; these will be adapted as necessary to the syntax of other search engines.

- MEDLINE (via Pubmed): National Library of Medicine's journal citation database of biomedical and life sciences journal articles.(10)
- EMBASE (via Ovid): Elsevier's international database with in depth coverage of pharmacology, pharmaceutical science, clinical research, basic biomedical science, veterinary science and extensive allied health topics.(11)
- SciELO: Electronic library covering a selected collection of Brazilian scientific journals, being developed by FAPESP - Fundação de Amparo à Pesquisa do Estado de São Paulo, in partnership with BIREME - the Latin American and Caribbean Center on Health Sciences Information.(12)
- CINAHL (via EBSCO): EBSCO's database indexing of the top nursing and allied health literature, covering nursing, biomedicine, health sciences librarianship, alternative/complementary medicine, consumer health and seventeen allied health disciplines.(13)
- PsycINFO (via EBSCO): A collection of behavioral and social science research, dissertations, and scholarly literature, including topics including neuroscience, business, nursing, law, and education. Coverage spans from the 17th century to the present, charting the evolution of psychology over time through peer-reviewed journals, books, and dissertations.(14)
- CUMED (via WHO Virtual Health Library): Bibliographic database developed by the National Medical Library and cooperating institutions of the national network of health information with records from Cuban medical and allied sciences published in Cuba or abroad.(15)
- LILACS (via WHO Virtual Health Library): Database of the Latin American and Caribbean of Health Sciences Information System.(15)
- IBECS (via WHO Virtual Health Library): Biographic Index on Health Sciences from Spain, a potential source of Spanish language publications from the Caribbean.(15)

The publication dates for the full review span a 10 year period- from January 1st, 2004 through December 31st, 2014. This period was chosen as it sandwiches the 2007 Port of Spain Declaration, and studies published more than 10 year ago were considered too old to inform current policy on social determinants. The search terms for the social determinants were guided by the extension of the PRISMA statement for reporting systematic reviews with a focus on health equity. (9) The statement recommends using the PROGRESS-Plus checklist, which includes place of residence, race/ethnicity/culture/language, occupation, gender/sex, religion, education, socio-economic status, social capital and any other possible factors. All other search terms were conceptualized through thorough broad research on depression studies to identify key indicators.

Study Selection

All studies selected for the systematic review will be downloaded into Endnote reference manager. (16) Study selection will be conducted in two steps by two reviewers:

1. Initial screening of titles and abstracts against the inclusion criteria to identify potentially relevant studies.
2. Secondary screening of the full-text studies identified as potentially relevant in the initial screening.

All studies will be reviewed by two reviewers. In instances where Step 1 is impossible to complete with only the title and abstract, the full-text is to be retrieved and screened as stated in Step 2. In instances where there is still poor clarity on whether to include a study, the study will be forwarded to an independent third party for consensus. The numbers of articles reviewed, selected, and excluded at each stage will be documented according to the flowchart depicted below in Figure 2.

Figure 2- Literature screening process according to the 2009 PRISMA flowchart template.(17)

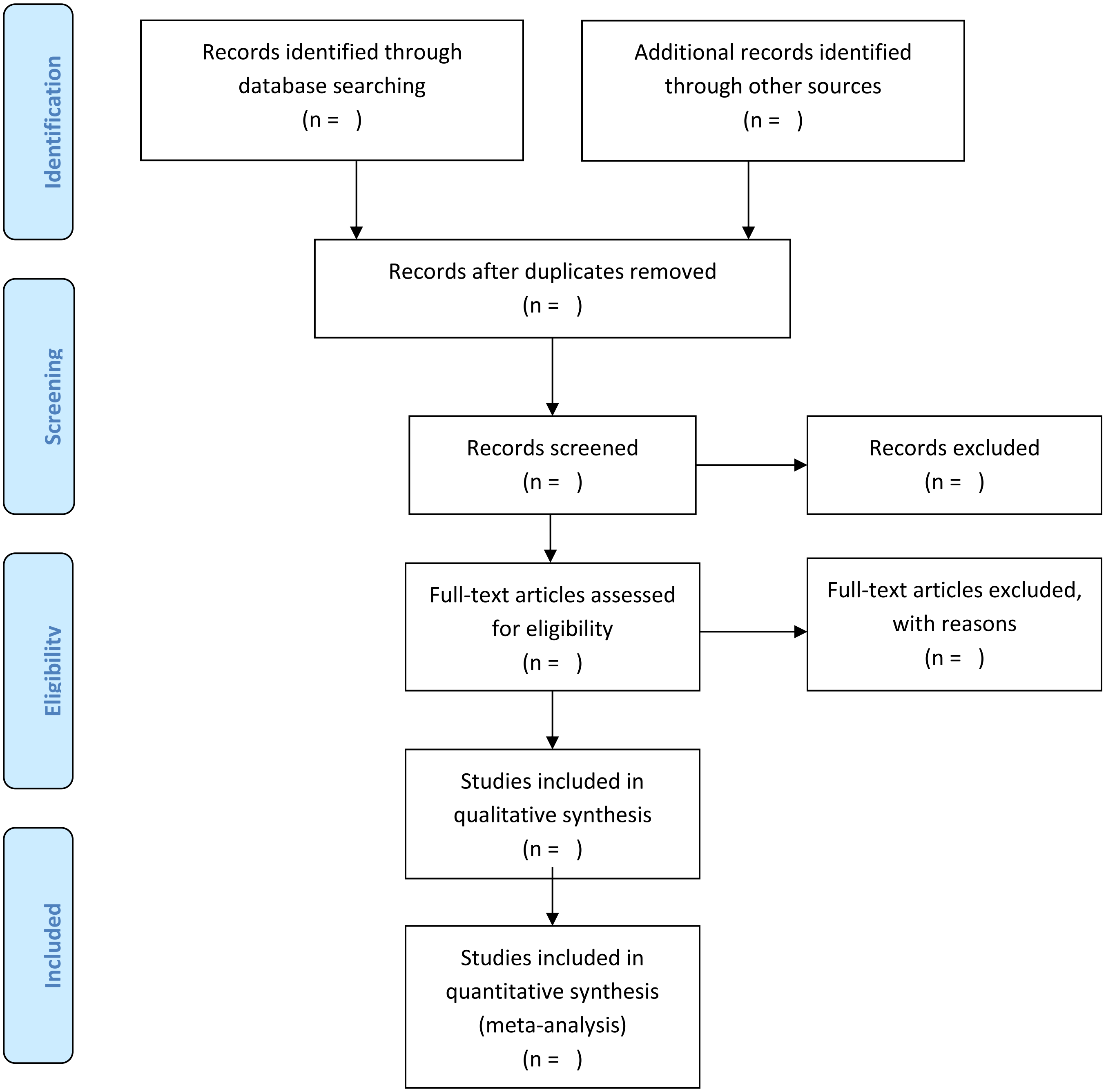

Data Extraction

Studies that pass both steps of the study selection process will be eligible for data extraction. Each full-text study will be independently data-extracted by two reviewers. Any discordance in data extraction will also be resolved by a third party reviewer. Data extraction forms have been created in RedCap software in order to manage the data.(18) Sample forms are illustrated in Appendix B. These forms are designed to extract key study characteristics and findings relevant to the primary research question. They have also been designed to enable an assessment of risk of bias inherent in each study (See appendix C for details on our risk of bias assessment). The content of the data abstraction form has been guided by the STROBE statement on reporting observational epidemiology and by the PRISMA statement on systematic reviews concerning health equity.(9,19)

Broadly, data items extracted from the included articles fall into one of the following information groups: basic study details (article title, author, publication year, study design, country/countries of data collection etc); risk factor details; disease details; adverse outcome details. The social determinants examined, tools/units of measurement, statistical techniques employed, results, confounders controlled, and assessment of risk of bias were depicted for each group. Should a study not have sufficient information required to fill out the data abstraction form to completion, that study will still be included in the review, but categorized as such.

Quality Assessment

Risk of bias will be assessed according a tool adapted from STROBE and Cochrane AROBAT-NRSi guidelines (see Appendix C).(19,20) Bias is to be assessed in 5 domains at the relationship level: confounding (might a relationship be affected by an unmeasured confounder?), participant selection (is the sample representative of the target population?), missing data (is the data reasonably complete?), outcome measurement (is a social determinant/disease endpoint appropriately measured?), selective reporting (is a relationship selectively reported?). To accommodate variability in depression survey tools, validation of the measurement tools and clinician involvement were considered for outcome measurement. Studies were classified as having serious, moderate, low, or unclear risk of bias. If a measurement tool was not validated or if a clinician did not conduct the survey, it was classified as high-risk under the outcome measurement domain. Two review authors (CB, MMM) made an independent judgement on the overall risk of bias in each included article, considering each domain as equally

important and the likely direction and magnitude of the classified bias from each domain. Discrepancies were discussed by the two reviewers to achieve consensus.

Data Analysis

The review is planned as a narrative synthesis of evidence, with meta-analysis of quantitative evidence restricted to studies classified as having a low and moderate risk of bias. Sensitivity analyses will be conducted with any relevant high-risk articles.

For the narrative synthesis, key study-level information will be summarized for all studies. Variable-level information will be summarized, focusing separately on associations between social determinants and risk factors, between social determinants and cancer frequency, then between social determinants and cancer outcomes. To summarize quantitative information, a random-effects meta-analysis will be used to recognize for the anticipated heterogeneity between studies. The I-squared value will be reported to quantify heterogeneity.(20) Rate ratios and odds ratios will be used to report social determinant differences in the outcome variables, depending on their type (rate ratio for rates or odds ratio for categorical outcomes).

Plans for Dissemination

It is expected that the findings from the scoping review will be submitted for peer-reviewed publication. In addition, findings will be shared at Caribbean regional meetings such as the Caribbean Health Research Council's annual meeting, as well as any relevant international Conferences.

References

1. World Health Organization. Depression [Internet]. World Health Organization. 2012 [cited 2014 Oct 27]. Available from: http://www.who.int/mediacentre/factsheets/fs369/en/

2. World Health Organization. Rio political declaration on social determinants of health [Internet]. Rio de Janeiro, Brazil: World Health Organization; 2011 Oct [cited 2014 Oct 27]. Available from: http://www.who.int/sdhconference/declaration/Rio_political_declaration.pdf?ua=1

3. Erik Blas, Anand Sivasankara Kurup, editors. Equity, social determinants, and public health programmes [Internet]. World Health Organization: World Press; 2010 [cited 2014 Oct 27]. 291 p. Available from: http://whqlibdoc.who.int/publications/2010/9789241563970_eng.pdf

4. World Health Organization. Global action plan for the prevention and control of noncommunicable diseases 2013-2020 [Internet]. WHO Press; 2013 [cited 2014 Jun 2]. Available from: http://apps.who.int/iris/bitstream/10665/94384/1/9789241506236_eng.pdf

5. Abel WD, Gibson R, Hickling FW. Depression: a major public healthproblem facing the Caribbean. West Indian Med J. 2005 Dec;54(6):353–4.

6. Institute for Health Metrics and Evaluation. GBD Compare [Internet]. Institute for Health Metrics and Evaluation. 2013 [cited 2016 Mar 13]. Available from: http://vizhub.healthdata.org/gbd-compare/arrow

7. Blas E, Kurup AS, editors. Equity, social determinants, and public health programmes [Internet]. World Health Organization: World Press; 2010 [cited 2014 Oct 27]. 291 p. Available from: http://whqlibdoc.who.int/publications/2010/9789241563970_eng.pdf

8. The global burden of mental disorders and the need for a comprehensive, coordinated response from health and social sectors at the country level [Internet]. Switzerland: World Health Organization; 2012 May [cited 2014 Nov 11]. (World Health Assembly Meeting). Report No.: A65/VR/9. Available from: http://apps.who.int/gb/ebwha/pdf_files/WHA65/A65_R4-en.pdf?ua=1

9. Welch V, Petticrew M, Tugwell P, Moher D, O'Neill J, Waters E, et al. PRISMA-Equity 2012 extension: reporting guidelines for systematic reviews with a focus on health equity. PLoS Med. 2012;9(10):e1001333.

10. U.S. National Library of Medicine. Fact Sheet MEDLINE, PubMed, and PMC (PubMed Central): How are they different? [Internet]. 2015 [cited 2015 Aug 13]. Available from: http://www.nlm.nih.gov/pubs/factsheets/dif_med_pub.html

11. Elsevier R&D Solutions. Embase Fact Sheet [Internet]. Elsevier B.V.; 2015 [cited 2015 Aug 13]. Available from: http://www.elsevier.com/__data/assets/pdf_file/0016/59011/R_D-Solutions_Embase_Fact-Sheet_DIGITAL.pdf

12. SciELO, FAPESP, BIRME. SciELO [Internet]. SciELO. n.d. [cited 2015 Aug 13]. Available from: http://www.scielo.br/

13. EBSCO. CINAHL Database [Internet]. 2015 [cited 2015 Aug 13]. Available from: https://health.ebsco.com/products/the-cinahl-database

14. EBSCO. PsycINFO [Internet]. EBSCO. 2015 [cited 2015 Mar 9]. Available from: https://www.ebscohost.com/academic/psycinfo

15. Centro Nacional de Información de Ciencias Médicas, Infomed. Bibliographic Databases [Internet]. BVS Cuba: Biblioteca Virtual en Salud. n.d. [cited 2015 Aug 13]. Available from: http://www.bvsite.sld.cu/php/level.php?lang=en&component=30&item=3

16. EndNote [Internet]. Philadephia: Thomson Reuters; 2014 [cited 2015 Nov 10]. Available from: www.endnote.com

17. Moher D, Liberati A, Tetzlaff J, Altman DG, Group TP. Preferred Reporting Items for Systematic Reviews and Meta-Analyses: The PRISMA Statement. PLOS Med. 2009 Jul 21;6(7):e1000097.

18. Harris PA, Taylor R, Thielke R, Payne J, Gonzalez N, Conde J. Research electronic data capture (REDCap) - A metadata-driven methodology and workflow process for providing translational research informatics support. J Biomed Inform. 2009;42(2):377–81.

19. von Elm E, Altman DG, Egger M, Pocock SJ, Gøtzsche PC, Vandenbroucke JP, et al. The Strengthening the Reporting of Observational Studies in Epidemiology (STROBE) statement: guidelines for reporting observational studies. J Clin Epidemiol. 2008 Apr;61(4):344–9.

20. A Cochrane risk of bias assessment tool: for non-randomized studies of interventions (ACROBAT-NRSI), version 1.0.0 [Internet]. The Cochrane Collaboration; 2014 Sep [cited 2015 Nov 11]. Available from: http://www.riskofbias.info

Appendices

Appendix A – Search Strategy for Pubmed Search Engine

Notes: Words that could be author names were restricted to non-author fields. Truncation (*) was not used in cases where the non-truncated word created a broader search because it triggers a MeSH term and automatically includes the pluralized form. Otherwise, both the truncated and non-truncated MeSH terms were used. Limits used: Human-only, date range = January 1, 2004 – December 31, 2014.

(Caribbean OR West Indies OR Leeward OR Windward OR Antilles OR Anguilla OR Antigua OR Aruba OR Barbuda OR Bahamas OR Barbados OR Barthelemy OR "St. Bartholomew" OR "Saint Bartholomew" OR Barts OR Belize OR Bermuda OR Bonaire OR Cayman OR Croix OR Cuba OR Curacao OR Dominica OR "Dominican Republic" OR Eustatius OR "Santo Domingo" OR "Saint Domingue" OR "St. Dominque" OR Grenada OR Guadeloupe OR Guyana OR Haiti OR Hispaniola OR Jamaica OR "St. John" OR "Saint John" OR "St. Thomas" OR "Saint Thomas" OR "St. Vincent" OR "Saint Vincent" OR "St. Martin" OR "Saint Martin" OR "St. Maarten" OR "Saint Maarten" OR Martinique[tw] OR Martinique[AD] OR Martinique [TA] OR Martinique [LID] OR Martinique [PL] OR Martinique [PUBN] OR "St. Nevis" OR "Saint Nevis" OR "St. Christopher and Nevis" OR "Saint Christopher and Nevis" OR "St. Lucia" OR "Saint Lucia" OR Kitts OR Montserrat OR "Puerto Rico" OR Grenadines OR "Virgin Islands" OR Saba OR Suriname OR Trinidad OR Tobago OR Tortola) AND (age OR gender OR education OR educat OR income OR wealth OR ethnic OR ethnic* OR race OR culture OR language OR occupation OR religion OR social class OR socioeconomic OR health social determinants OR social determinant* OR social capital OR residence OR medical geography OR health service OR health service* OR health equity OR disparit* OR medical sociology OR prejudice OR health insurance OR health gradient OR health gap OR vulnerable populations OR continental population groups OR Arawak* OR Amerindian* OR carib OR caribs OR taino* OR ethnic groups OR social conditions OR urban OR rural OR urban health OR urban population OR rural health OR rural population OR social position OR poverty OR wealth OR rich[tw] OR poor OR social support OR discriminat* OR differenti* OR globaliz* OR globalis* OR urbanization OR urbaniz* OR urbanis* OR westerniz* OR westernis*) AND ("mental illness" OR mental disorders OR depressive disorder OR depress* OR "disruptive mood dysregulation disorder" OR "premenstrual dysphoric disorder" OR dysthymia OR dysphoria OR hopeless* OR mood* OR agitat* OR irrita* OR sad OR sadness OR bereave* OR HRSD OR HDRS OR HAM-D OR MADRS OR BDI OR QIDS OR suicid* OR para-suicide OR parasuicide OR self-mutilat* OR self-harm)*

Appendix B – Redcap Data Extraction Forms

Depression: Basic Data Form

Record ID	DP 36
Abstractor	⊗ CB<br>◯ SH<br>◯ NSG
Article title	Some favouring factors of the neurotic depression in hospitalized elderly<br>(Title of paper)
Author	Perez<br>(Last name of first author)
Publication year	2012<br>(Year of article publication)
Study name	______________________<br>(Name of the study which produced the data)
Does this article describe at least one disease measurement or complication by one of the selected social determinants?	⊗ Yes<br>◯ No
Study size	81<br>(Final number of participants in the study)
Study design	◯ Case report<br>◯ Case control<br>◯ Cohort-prospective<br>◯ Cohort-retrospective<br>⊗ Cross-sectional<br>◯ RCT<br>◯ Qualitative design<br>◯ Registry-based study<br>◯ Other
Does the article examine data from a Caribbean territory?	⊗ Yes<br>◯ No
Does it meet criteria for review?	⊗ Yes<br>◯ No<br>◯ Awaiting assessment
Age range	60-89<br>(State the age range of the study participants)
Number of Caribbean countries	1
Name of first country	Cuba
Names of extra-regional countries	______________________
Beginning of study period	15-01-2010<br>(State the beginning of the study period. If no day given assume 15th day of month given. If no month given assume mid-year start at July 1.)
End of study period	15-12-2010<br>(State the end of the study period. If no day given assume 15th day of month given. If no month given assume mid-year start at July 1.)
Response rate	______________________

Comments on response rate

____
(Provide comments on the methods used to calculate the response rate, if not stated please indicate "methods not stated")

Study base

- ☐ population
- ☐ region/community
- ☒ health facility
- ☐ school
- ☐ workplace
- ☐ church
- ☐ other

Depression: Disease Burden Form

Record ID	DP 36
Disease criteria	⊗ Yes<br>○ No<br>(Does this paper examine the distribution of the relevant disease by a selected social determinant?)
Measurement of depression	☒ prevalence<br>☐ prevalent cases<br>☐ incidence<br>☐ incident cases
Check all social determinants studied in relation to prevalence of depression	☒ gender<br>☐ occupation<br>☒ income<br>☐ education<br>☐ race/ethnicity<br>☐ language<br>☐ culture<br>☐ religion<br>☐ residence (geography)<br>☐ social capital<br>☐ social support<br>☐ physical infrastructure<br>☐ healthcare systems<br>☐ social household structure<br>☐ marital status<br>☒ age<br>☐ other
State the number of social determinants described by depression prevalence	3
Was gender measured objectively or subjectively?	○ Objectively<br>⊗ Subjectively<br>○ Both<br>○ Not stated
Was gender measured continuously or categorically?	○ Continuous<br>⊗ Categorical
State the categories of gender	male, female
Describe the measurement tool used to determine gender	______________________
Was income measured objectively or subjectively?	○ Objectively<br>⊗ Subjectively<br>○ Both<br>○ Not stated
Was income measured continuously or categorically?	○ Continuous<br>⊗ Categorical
State the categories of income	good, regular, poor
Describe the measurement tool used to determine income	______________________

Was age measured objectively or subjectively?	○ Objectively<br>⊗ Subjectively<br>○ Both<br>○ Not stated
Was age measured continuously or categorically?	○ Continuous<br>⊗ Categorical
State categories of age	60-69, 70-79, 80-89
Describe measurement tool used to determine age	__________________
Describe measurement tool used to determine SD other3	__________________
Was depression prevalence measured objectively or subjectively?	○ Objectively<br>⊗ Subjectively<br>○ Both<br>○ Not stated
Was depression prevalence measured continuously or categorically?	○ Continuous<br>⊗ Categorical
Describe measurement tool used to determine depression prevalence	Geriatric Depression Scale was used for data collection which was reliability-tested by Cronbach's alpha.
Choose the statistical technique used to analyse the relationship between SD and depression prevalence	☐ Fisher's exact test<br>☐ Chi2 test<br>☐ Chi2 test for trend<br>☐ McNemar's test<br>☐ Independent T-test<br>☐ Paired T-test<br>☐ Mann-Whitney U test<br>☐ ANOVA<br>☐ Simple logistic regression<br>☐ Multiple logistic regression<br>☐ Simple linear regression<br>☐ Multiple linear regression<br>☐ Log rank test<br>☐ Cox regression<br>☐ CI around means<br>☐ CI around proportions<br>☒ Other
Other statistical techniques used	proportion
Describe the main result between the social determinant and depression prevalence	Gender: The proportions of are as follows - males (7%), females (93%). Age-stratified proportions are as follows (males, females) - 60- 69 (9%, 91%), 70-79 (7%, 93%), 80-89 (N/A). These were calculated from Table 1.<br><br>Income: The proportions are a follows - good (4.9%, regular (12.4%), poor (82.7%).<br><br>Age: The proportions of are as follows - 60- 69 (54.3%), 70-79 (33.3%), 80-89 (12.4%). Gender-stratified proportions are as follows (60-69, 70-79, 80-89) - males (66.7%, 33.3%, NA), females (53.3%, 33.3%, 13.4%). These were calculated from Table 1.
Was age adjusted as a confounder for the relationship between depression prevalence and SD?	⊗ Yes<br>○ No

Was gender adjusted as a confounder for the relationship between depression prevalence and SD?	⊗ Yes<br>◯ No
List all other potential confounders controlled for in the relationship between depression prevalence and SD	__________________________________
Assess the quality of the relationship between depression prevalence and SD	⊗ High risk of bias<br>◯ Medium risk of bias<br>◯ Low risk of bias<br>◯ Unclear
Provide a rational for your bias assessment on the relationship between depression prevalence and SD	Not population based
State the categories of age	__________________________________

Depression: Outcomes Form

Record ID	DP 36
Outcome criteria	⊗ Yes<br>○ No<br>(Does this paper examine the distribution of a relevant depression outcome by a selected social determinant?)
Depression outcome	☐ depression score<br>☒ suicide ideation<br>☐ para-suicide<br>☐ suicide<br>☐ all-cause mortality
Check all social determinants studied in relation to suicide ideation	☐ gender<br>☐ occupation<br>☐ income<br>☒ education<br>☐ race/ethnicity<br>☐ religion<br>☐ residence (geography)<br>☐ social capital<br>☐ social support<br>☐ physical infrastructure<br>☐ healthcare systems<br>☐ social household structure<br>☐ marital status<br>☐ age<br>☐ other
State the total number of social determinants examined by suicide ideation	1
Was education measured objectively or subjectively?	○ Objectively<br>⊗ Subjectively<br>○ Both<br>○ Not stated
Was education measured continuously or categorically?	○ Continuous<br>⊗ Categorical
State the categories of education	primary school, secondary school
Describe measurement tool used to determine education	______________________________
Was suicide ideation measured objectively or subjectively?	○ Objectively<br>⊗ Subjectively<br>○ Both<br>○ Not stated
Was suicide ideation measured continuously or categorically?	○ Continuous<br>○ Categorical
Describe measurement tool used to determine suicide ideation	Geriatric Depression Scale was used for data collection which was reliability-tested by Cronbach's alpha.

Choose the statistical technique used to analyse the relationship between SD and suicide ideation	☐ Fisher's exact test<br>☐ Chi2 test<br>☐ Chi2 test for trend<br>☐ McNemar's test<br>☐ Independent T-test<br>☐ Paired T-test<br>☐ Mann-Whitney U test<br>☐ ANOVA<br>☐ Simple logistic regression<br>☐ Multiple logistic regression<br>☐ Simple linear regression<br>☐ Multiple linear regression<br>☐ Log rank test<br>☐ Cox regression<br>☐ CI around means<br>☐ CI around proportions<br>☒ Other
Other statistical techniques used	proportion
Describe the main result between the social determinants and suicide ideation	Education: Proportions of suicide ideation in females are as follows - primary school (74.1%), secondary school (12.4%)
Was age adjusted as a confounder for the relationship between suicide ideation and SD?	○ Yes<br>○ No
Was gender adjusted as a confounder for the relationship between suicide ideation and SD?	⊗ Yes<br>○ No
List all other potential confounders controlled for in the relationship between suicide ideation and SD	______________________
Assess the quality of the relationship between suicide ideation and SD	⊗ High risk of bias<br>○ Medium risk of bias<br>○ Low risk of bias<br>○ Unclear
Provide a rational for your bias assessment on the relationship between suicide ideation and SD	Not population based

Appendix C – Risk of Bias Assessment Tool

Version 3.1

12-Dec-2015

This tool is a simplification of the Cochrane ACROBAT-NRSI tool, with adaptations to account for the fact that our systematic reviews do not include non-randomised studies of interventions (NRSI). The types of non-randomised studies that are assessed using this adapted tool are observational studies of any design that report relationships between a social determinant and known risk factors for a specific disease, disease frequency (such as incidence or prevalence), or disease outcomes (such as survival or mortality).

ACROBAT-NRSI is based on the Cochrane Risk of Bias (RoB) tool for randomized trials, which was launched in 2008 and modified in 2011. As in the tool for randomized trials, risk of bias is assessed within specified bias domains, and review authors document the information on which judgements are based.

The focus of this RoB tool is on internal validity. We define bias as a tendency for study results to differ systematically from the results expected from a study of the same design, conducted on the same participant group, and that had no flaws in its conduct. Such bias is distinct from issues of generalizability (applicability) to types of individual who were not included from the study.

The domains of bias used in this adapted RoB tool have the following meaning:

Bias due to confounding	A confounding variable is a prognostic factor that may partly predict whether a participant has a particular value of a social determinant.<br><br>**Example.** For the relationship between level of education (a social determinant) and prostate cancer prevalence (a measure of disease frequency), age and sex would be important confounding factors as both age and sex would also be expected to influence a person's level of education.

Bias in participant selection	Selection bias occurs when some eligible participants are excluded in a way that leads to the association between the social determinant and the outcome differing from the association that might have been observed in the absence of these exclusions.<br><br>**Example.** For the relationship between level of education (a social determinant) and prostate cancer (a measure of disease frequency), participant non-selection may have been related to level of education, with (for example) those with lower levels of education less likely to participant in the study.
Bias due to missing data	Missing data may arise, among other reasons, through attrition (loss to follow up), missed appointments, incomplete data collection and by participants being excluded from analysis by primary investigators. In NRS, data may be missing for social determinants, for disease risk factors, frequency or outcomes, or for other variables involved in the analysis or a combination of these.<br><br>A general rule for consideration of bias due to missing data is that we should assume that an analysis using the data we intended to collect (were they available) would produce an unbiased effect estimate, so that biases might reasonably be introduced by any missing data.
Bias in measurement of outcomes	Bias may be introduced if social determinants, disease risk factors, disease frequency, or disease outcomes are misclassified or measured with error.
Bias in selection of reported results	Selective reporting is the failure to report, or partial reporting of relationships between social determinants and either risk factors, disease frequency, or disease outcomes that were measured and analysed. Selective reporting might be (a) selective reporting of a particular outcome measurement from multiple measurements; (b)

	selective reporting of a particular analysis from multiple analyses of a specific outcome measurement; and (c) selective reporting of a subset of the participants.

DOMAIN 1: Confounding.

Table A. Questions for each relationship

(one table to be completed for each relationship)

**Relationship Description:**					
	No	Possibly No	Possibly Yes	Yes	No Info
1.1 Is confounding of the relationship between the social determinant and the disease endpoint unlikely in this study?					
1.2. Did the authors use an appropriate analysis method that adjusted for all the critically important confounding domains?					
1.3. Were confounders that were adjusted for measured validly and reliably by the variables available in this study?					

Table B. How to Judge and Apply Risk of Bias to each relationship

Low Risk of Bias	No confounding expected
Moderate Risk of bias	Confounding expected, all known critically important confounding domains appropriately measured and adjusted for; **AND** Reliability and validity of measurement of critically important domains were sufficient that we do not expect serious residual confounding.

Serious Risk of Bias	At least one known critically important domain not appropriately measured, or not adjusted for; **OR** Reliability or validity of measurement of a critically important domain was low enough that we expect serious residual confounding.
Unclear Risk of Bias	No information on whether confounding might be present.

Table C. Risk of Bias Judgement for each relationship

(Add rows for >5 relationships)

**Relationship**	**Confounding**	**Comment**
R1		
R2		
R3		
R4		
R5		

DOMAIN 2: Bias in Selection of Participants to Study.

Table A1. Cross-sectional & Registry Studies - questions for each relationship

(one table to be completed for each relationship)

**Relationship Description:**					
	No	Possibly No	Possibly Yes	Yes	No Info
2.1 Representative of target population *					
	<50%	50 to 75%	75% to 90%	>90%	No Info
2.2 Response rate **					

*** Target population need not be general population. Also, registry-based studies will be examined as cross-sectional studies; the quality of the registry will be assessed via Question 2.1 only.**

**** Not applicable to registry-based studies.**

Table A2. Cohort Studies - questions for each relationship

(one table to be completed for each relationship)

**Relationship Description:**					
	No	Possibly No	Possibly Yes	Yes	No Info
2.3 Representative of target population *					
	>50%	25% to 50%	10% to 25%	<10%	No Info
2.4 Attrition rate					

*** Target population need not be general population.**

Table A3. Case-Control Studies - questions for each relationship

(one table to be completed for each relationship)

**Relationship Description:**					
	No	Possibly No	Possibly Yes	Yes	No Info
2.5 Cases and Controls taken from same or similar population					

Table B. How to Judge and Apply Risk of Bias to each relationship

Low Risk of Bias	**Cross-sectional:** Representative of target population **AND** response rate >75%<br><br>**Cohort:** Representative of target population **AND** Attrition rate <10%<br><br>**Case-Control:** Cases and controls from same or similar populations
Moderate Risk of bias	**Cross-sectional:** Representative of target population **AND** response rate 50%-75%<br><br>**Cohort:** Representative of target population **AND** Attrition rate 25-50%

	**Case-Control:** Cases and controls possibly from same or similar populations
Serious Risk of Bias	**Cross-sectional:** Not representative of target population **OR** response rate <50%<br><br>**Cohort:** Not representative of target population **OR** Attrition rate >50%<br><br>**Case-Control:** Cases and controls possibly not or not from same or similar populations
Unclear Risk of Bias	No information on whether confounding might be present.

Table C. Risk of Bias Judgement for each relationship

(Add rows for >5 relationships)

**Relationship**	**Selection of Participants**	**Comment**
R1		
R2		
R3		
R4		
R5		

DOMAIN 3: Bias due to missing data.

Table A. Questions for each relationship

(one table to be completed for each relationship)

**Relationship Description:**					
	>20%	15% to 20%	10% to 15%	<10%	No Info
3.1 Exclusion of potentially eligible participants because of missing data					
	No	Possibly No	Possibly Yes	Yes	No Info
3.2 Were appropriate statistical methods used to account for missing data					

Table B. How to Judge and Apply Risk of Bias to each relationship

Low Risk of Bias	Data were reasonably complete (<10% missing) **OR** appropriate statistical analyses used to account for missing data
Moderate Risk of bias	Missing data (10-20%) **AND** missing data not addressed in the analysis
Serious Risk of Bias	Missing data (>20%) regardless if addressed in the analysis.
Unclear Risk of Bias	No information on whether confounding might be present.

Table C. Risk of Bias Judgement for each relationship

(Add rows for >5 relationships)

Relationship	Missing Data	Comment
R1		
R2		
R3		
R4		
R5		

DOMAIN 4: Bias in measurement of outcomes.

Table A. Questions for each relationship

(one table to be completed for each relationship)

**Relationship Description:**					
	No	Possibly No	Possibly Yes	Yes	No Info
4.1 Social determinant is appropriately measured? *					
4.2 Risk factor / disease frequency / disease outcome measured objectively **					

*** Social determinants measured via self-report would likely be listed as "Possibly Yes"**

**** Risk factors which are unlikely to be measured objectively (alcohol, physical activity), and are instead measured via self-report, can be considered as "possibly yes"**

Table B. How to Judge and Apply Risk of Bias to each relationship

Low Risk of Bias *	Social determinant is appropriately measured (yes or possibly yes) <u>**AND**</u> risk factor / disease frequency / disease outcome is objectively measured (yes / possibly yes)

Moderate Risk of bias	Social determinant **not** appropriately measured (no or possibly no) **AND** Risk factor / disease frequency / disease outcome is objectively measured (yes / possibly yes)
Serious Risk of Bias	Social determinant **not** appropriately measured (no or possibly no) **AND** risk factor / disease frequency / disease outcome **not** objectively measured (no or possibly no)
Unclear Risk of Bias	No information on whether confounding might be present.

*** EXCEPTION: If social determinant and risk factor are measured via self-report out of necessity (eg: alcohol consumption), then risk of bias is considered as moderate, not low.**

Table C. Risk of Bias Judgement for each relationship

(Add rows for >5 relationships)

**Relationship**	**Measurement of Outcomes**	**Comment**
R1		
R2		
R3		
R4		
R5		

DOMAIN 5: Bias in selection of the reported results.

Table A. Questions for each relationship

(one table to be completed for each relationship)

**Relationship Description:**					
	No	Possibly No	Possibly Yes	Yes	No Info
5.1 From the study report, do the results section and figures/tables					

reflect the data and analyses described in the study methods *					
5.2 Is there evidence of multiple endpoints within the same endpoint domain **					
5.3 Is there evidence of multiple analyses for a single social determinant-endpoint relationship ***					

“* If paper describes the methods section poorly, this would likely be listed as “No Info”

**** An example of this might be BMI and Waist Circumference, both used as measures of adiposity. Also, this does not refer to the abstractors’ own constructs (eg: if article lists maternal age, maternal education as single independent variables, and abstractor categorizes all as proxies of SES)**

***** This question relates directly to 5.2 only, referring to multiple analyses of a single endpoint domain with multiple endpoints examined differently. An example of this might be univariate, then adjusted analyses for the same relationship.**

Table B. How to Judge and Apply Risk of Bias to each relationship

Low Risk of Bias	There is clear evidence (through examination of a protocol or statistical analysis plan) that all reported results correspond to all intended outcomes and analyses.
Moderate Risk of Bias	Relationship and analyses are not consistent with a stated a priori plan, but there is no absolute evidence of selective endpoint use or of multiple analyses for the same relationship.
Serious Risk of Bias	Relationship and analyses are not consistent with an a priori plan **OR** there is absolute evidence (“Yes” answers only) of selective endpoint use **OR** of multiple analyses for the same relationship
Unclear Risk of Bias	No information on whether confounding might be present.

Table C. Risk of Bias Judgement for each relationship

(Add rows for >5 relationships)

**Relationship**	**Selected Reporting**	**Comment**
R1		
R2		
R3		
R4		
R5		

ALL DOMAINS: Summary Risk of Bias Table.

Risk of Bias Judgement for all domains combined (Add rows for >5 relationships)

No definitive criteria for determining the Overall RoB as is subjectively based on the qualitative feel of the paper. A general rule of thumb might be that the Overall RoB is most likely to be the same as the worst classification of 5 Domains, but with exceptions.

**Relationship**	**Domain 1: Confounding**	**Domain 2: Selection of Participants**	**Domain 3: Missing Data**	**Domain 4: Measurement of Outcomes**	**Domain 5: Selected Reporting**	**OVERALL RoB**
**R1**						
**R2**						
**R3**						
**R4**						
**R5**						
