# Supplementary material for: Social determinants of depression and suicidal behaviour in the Caribbean: a systematic review
Source: BMC Public Health. 2017 Jun 15;17:577. doi: 10.1186/s12889-017-4371-z (PMC5472962; doi:10.1186/s12889-017-4371-z)
Supplement: Supplementary file 6 — Supplementary Narrative of Results of Inequality Relationships, which describes additional results for remaining social determinants not discussed narratively in the main paper. (DOCX 54 kb) [file 12889_2017_4371_MOESM6_ESM.docx]

Supplementary Narrative Results of Inequality Relationships

Ethnicity

Ethnicity was examined in 11 inequality relationships across 8 articles: prevalence (n=4), depression score (n=2), suicidal ideation (n=1), parasuicide (n=2), suicide (n=2). No studies were found examining language or culture as proxies for ethnicity. All 11 relationships were classified as having serious risk of bias. All but one study originated from Trinidad and Tobago. Studies examining depression prevalence showed no showed no association with ethnicity.(1–4) The same applies to depression score, except one finding that female of direct East Indian descent showed lower scores than mixed African-East Indian females.(5) Likewise, the one study examining suicidal ideation found Indians to have higher mean scores than Africans (x=28.1 vs x=23.5, p<0.05).(6) While parasuicide studies showed no association with ethnicity, one study examining suicide reported East Indian ethnicity to be correlated with higher rates of suicide than other races (r_s_=0.72).(7)

Income

Income was examined in 15 inequality relationships across 12 articles: prevalence (n=6), depression score (n=3), suicidal ideation (n=2), parasuicide (n=3), suicide (n=1). Among these, 5 were classified as having moderate risk of bias, 9 as having serious risk of bias, and 1 as having unclear risk of bias. Studies examining depression prevalence and depression score found higher figures among persons with less income, also when perceived economic situation, number of household possessions, and school type are used as proxies for income.(2,8–13) Yet no associations were found between income were found between income and suicidal ideation or parasuicide.(6,14,15) Suicide, however, was shown to be more common among persons living in areas of lower income in Trinidad (R=0.84, p<0.02).(7)

Occupation

Occupation was examined in 13 inequality relationships across 11 articles: prevalence (n=6), depression score (n=1), suicidal ideation (n=1), parasuicide (n=4), suicide (n=1). Among these, 3 were classified as having moderate risk of bias, 9 as having serious risk of bias, and 1 as having unclear risk of bias. While no trend was seen across between depression frequency and occupation status/levels(1,3,9,12,16,17), unemployed persons tended to have slightly higher depression scores and more suicidal ideation (OR 1.4, 95%CI 1.0-1.8) than employed persons in Jamaica and Puerto Rico respectively.(17,18) Similarly, studies examining parasuicide showed a trend of higher parasuicide occurrences among persons who are unemployed or in lower-level occupations.(14,18–20) In contrast, suicide was reported to be committed in Jamaica more frequently among by persons holding more professional occupations rather than those holding lower-level positions such as clerks, agricultural workers, and shop workers.(21)

Religion

Religion was examined in 7 inequality relationships across 5 articles: prevalence (n=2), depression score (n=1), suicidal ideation (n=2), parasuicide (n=2). All 7 relationships were classified as having serious risk of bias. Overall, an inverse relationship was seen between religion and the four outcome variables. Though no association was seen across religion(4), a higher prevalence of depression and depression score was found in Trinidad adolescents who participate in fewer religious behaviours, such as prayer (19% vs 11% prevalence; x=59 vs x=64 depression score) and attendance at religious institution (15% vs 13% prevalence; x=62 vs x=61 depression score) than those who participate more often.(2) The same applies for a study examining suicidal ideation in Trinidad adolescents. Within the past six months, those who had attended a religious institution >7 times reported lower ideation than those who did not attend a religious institution at all (x=26 vs x=31) and those who had prayed with their family >7 times had lower ideation than those who did not pray at all (x=23 vs x=32).(6) Parasuicide was found to occur more in Trinidad adolescents who pray less with family (x=11% vs x=5%), and in Jamaican adolescents who consider themselves as not religiously affiliated (OR 2.39, 95%CI 0.81-7.01).(6,22)

Crowding & Physical Infrastructure
Crowding was examined in 1 inequality relationship in 1 article which was classified as having high risk of bias. Researchers reported that, in an urban area of Jamaica, the prevalence of depression was higher among persons living in crowded homes (22.5%) than those living uncrowded homes (18.2%).(12) This disparity was more apparent among males (2.6% difference) than females (0.8% difference). Physical infrastructure was examined in 2 inequality relationships in 2 articles: prevalence (n=1), depression score (n=1). Both relationships were classified as having moderate risk of bias. In an urban area of Jamaica, those living in low quality neighbourhoods (poorer house conditions, noise level, air quality, streets/sidewalks/yards) were 1.92 (95%CI 1.05-3.52) and 1.40 (95%CI 0.74-2.64) times more likely to report depressive symptoms than those living in high quality neighbourhoods.(12) Likewise, depression scores of adolescents in Jamaica and St. Kitts and Nevis were found to be predicted by neighbourhood disorder (regression coefficient = 1.18 and 0.52 respectively, p<0.05).(23)

Social Capital & Social Support

Social capital was examined in 10 inequality relationships across 9 articles: prevalence (n=3), depression score (n=2), suicidal ideation (n=3), parasuicide (n=2). Among these, 4 were classified as having moderate risk of bias, 4 as having serious risk of bias, and 2 as having unclear risk of bias. Social support was examined in 3 inequality relationships in 3 articles: prevalence (n=1), suicidal ideation (n=1), parasuicide (n=1). The prevalence of depression was found to be higher among persons with less diversity of links and who live in communities with negative attributes.(8,12,24) One of the two studies examining depression score showed a relationship, reporting higher scores in adolescents living in areas with less reported social cohesion and more crime.(23) Likewise, suicidal ideation was higher among adolescents with less close friends, those who spend free time alone rather than with friends/family, and those living in communities of more crime.(22,25,26) Adults in Haiti without someone to care for them if they are sick reported more suicidal ideation (OR 5.5, 95%CI 1.1-28.6).(13) Parasuicide also occurred more often in persons with less social contact or who spends free time alone rather than with friends/family.(14,22) No associations were found for social support and depression prevalence and parasuicide.(8,22)

Social Household Structure

Social household structure was examined in 14 inequality relationships across 13 articles: prevalence (n=7), depression score (n=2), suicidal ideation (n=1), parasuicide (n=4). Among these, 3 were classified as having moderate risk of bias, 9 as having serious risk of bias, and 2 as having unclear risk of bias. All studies examining adolescents found a higher depression prevalence, depression scores, suicidal ideation, and parasuicide among those living in reconstituted/blended families, as opposed to intact or single-parent families.(2,4,6,24,27,28) Among elderly Cubans, a gender difference was found for depression prevalence: females living in extended families (versus nuclear or partner-only family) and males living with their partner only (versus nuclear or extended family) showed the lowest prevalence of depression.(8) Among Jamaican adults, a higher depression prevalence was reported in those with fewer children and higher depression scores were reported by those living in households with fewer members(10); parasuicide was also reportedly higher among Martinique adults who live alone.(14,20)

# References

1. Maharaj RG, Reid SD, Misir A, Simeon DT. Depression and its associated factors among patients attending chronic disease clinics in southwest Trinidad. West Indian Med J. 2005 Dec;54(6):369–74.

2. Maharajh HD, Ali A, Konings M. Adolescent depression in Trinidad and Tobago. Eur Child Adolesc Psychiatry. 2006 Feb;15(1):30–7.

3. Maharaj RG. Depression and the nature of Trinidadian family practice: a cross-sectional study. BMC Fam Pract. 2007;8:25.

4. Maharaj RG, Alli F, Cumberbatch K, Laloo P, Mohammed S, Ramesar A, et al. Depression among adolescents, aged 13-19 years, attending secondary schools in Trinidad: prevalence and associated factors. West Indian Med J. 2008 Sep;57(4):352–9.

5. Nichols SD, Dookeran SS, Ragbir KK, Dalrymple N. Body image perception and the risk of unhealthy behaviours among university students. West Indian Med J. 2009 Nov;58(5):465–71.

6. Ali A, Maharajh HD. Social predictors of suicidal behaviour in adolescents in Trinidad and Tobago. Soc Psychiatry Psychiatr Epidemiol. 2005 Mar;40(3):186–91.

7. Hutchinson G. Variation of homicidal and suicidal behaviour within Trinidad and Tobago and the associated ecological risk factors. West Indian Med J. 2005 Oct;54(5):319–24.

8. Sicotte M, Alvarado BE, León E-M, Zunzunegui M-V. Social networks and depressive symptoms among elderly women and men in Havana, Cuba. Aging Ment Health. 2008 Mar;12(2):193–201.

9. Suau GM, Normandia R, Rodriguez R, Romaguera J, Segarra L. Depressive symptoms and risk factors among perimenopausal women. P R Health Sci J. 2005 Sep;24(3):207–10.

10. Monroe CE, Affuso O, Martin MY, Aung M, Crossman L, Jolly PE. Correlates of symptoms of depression and anxiety among clinic patients in western Jamaica. West Indian Med J. 2013 Jul;62(6):533–42.

11. Pérez G, Nora R, Sosa Zamora M, Reyes C, Luís J, Mojena Orue D, et al. Algunos factores favorecedores de la depresión neurótica en longevos hospitalizados. MEDISAN. 2012 Sep;16(9):1366–72.

12. Mullings JA, McCaw-Binns AM, Archer C, Wilks R. Gender differences in the effects of urban neighborhood on depressive symptoms in Jamaica. Rev Panam Salud Pública Pan Am J Public Health. 2013 Dec;34(6):385–92.

13. Wagenaar BH, Hagaman AK, Kaiser BN, McLean KE, Kohrt BA. Depression, suicidal ideation, and associated factors: a cross-sectional study in rural Haiti. BMC Psychiatry. 2012;12:149.

14. Slama F, Dehurtevent B, Even J-D, Charles-Nicolas A, Ballon N, Slama R. Characteristics of a French African Caribbean Epidemiological Psychiatric Sample with a History of Suicide Attempt. Suicide Life Threat Behav. 2008 Dec 1;38(6):720–7.

15. Bandiera FC, Ramirez R, Arheart KL, Canino G, Goodwin RD. Asthma and suicidal ideation and behavior among Puerto Rican older children and adolescents. J Nerv Ment Dis. 2013 Jul;201(7):587–91.

16. Alvarado BE, Zunzunegui MV, Béland F, Sicotte M, Tellechea L. Social and gender inequalities in depressive symptoms among urban older adults of latin america and the Caribbean. J Gerontol B Psychol Sci Soc Sci. 2007 Jul;62(4):S226-236.

17. Lowe GA, Lipps GE, Young R. Factors associated with depression in students at The University of the West Indies, Mona, Jamaica. West Indian Med J. 2009 Jan;58(1):21–7.

18. Vera M, Reyes-Rabanillo ML, Huertas S, Juarbe D, Pérez-Pedrogo C, Huertas A, et al. Suicide ideation, plans, and attempts among general practice patients with chronic health conditions in Puerto Rico. Int J Gen Med. 2011;4:197–205.

19. Veranes MC, Sánchez AG, Alvarez G, María L, Laera YM, Naranjo JN. Tentativa de suicidio en la población mayor de 15 años durante el bienio 2011-2012. MEDISAN. 2013 Oct;17(10):6072–9.

20. Slama F, Merle S, Ursulet G, Charles-Nicolas A, Ballon N. Prevalence of and risk factors for lifetime suicide attempts among Caribbean people in the French West Indies. Psychiatry Res. 2011 Dec 30;190(2–3):271–4.

21. Abel WD, James K, Bridgelal-Nagassar R, Holder-Nevins D, Eldemire H, Thompson E, et al. The epidemiology of suicide in Jamaica 2002-2010: rates and patterns. West Indian Med J. 2012 Aug;61(5):509–15.

22. Kukoyi OY, Shuaib FM, Campbell-Forrester S, Crossman L, Jolly PE. Suicidal ideation and suicide attempt among adolescents in Western Jamaica: a preliminary study. Crisis. 2010;31(6):317–27.

23. Lowe GA, Lipps G, Gibson RC, Halliday S, Morris A, Clarke N, et al. Neighbourhood Factors and Depression among Adolescents in Four Caribbean Countries. PLOS ONE. 2014 Apr 23;9(4):e95538.

24. Abel WD, Bailey-Davidson Y, Gibson RC, Martin JS, Sewell CA, James S, et al. Depressive symptoms in adolescents in Jamaica. West Indian Med J. 2012 Aug;61(5):494–8.

25. Rudatsikira E, Muula AS, Siziya S. Prevalence and associated factors of suicidal ideation among school-going adolescents in Guyana: results from a cross sectional study. Clin Pract Epidemiol Ment Health CP EMH. 2007 Aug 23;3:13.

26. Abel WD, Sewell C, Martin JS, Bailey-Davidson Y, Fox K. Suicide ideation in Jamaican youth: sociodemographic prevalence, protective and risk factors. West Indian Med J. 2012 Aug;61(5):521–5.

27. Ekundayo OJ, Dodson-Stallworth J, Roofe M, Aban IB, Kempf MC, Ehiri JE, et al. Prevalence and correlates of depressive symptoms among high school students in Hanover, Jamaica. Sci World J. 2007;7:567–76.

28. López JN, Vázquez MM. Factores de riesgo de intento suicida en adolescentes. MEDISAN. 2010 Apr;14(3):0–0.
